# Differentiation of white matter histopathology using b-tensor encoding and machine learning

**DOI:** 10.1371/journal.pone.0282549

**Published:** 2023-06-23

**Authors:** Ricardo Rios-Carrillo, Alonso Ramírez-Manzanares, Hiram Luna-Munguía, Mirelta Regalado, Luis Concha

**Affiliations:** 1 Instituto de Neurobiologia, Universidad Nacional Autónoma de Mexico, Querétaro, México; 2 Centro de Investigación en Matemáticas A.C., Guanajuato, México; University of Rochester, UNITED STATES

## Abstract

Diffusion-weighted magnetic resonance imaging (DW-MRI) is a non-invasive technique that is sensitive to microstructural geometry in neural tissue and is useful for the detection of neuropathology in research and clinical settings. Tensor-valued diffusion encoding schemes (b-tensor) have been developed to enrich the microstructural data that can be obtained through DW-MRI. These advanced methods have proven to be more specific to microstructural properties than conventional DW-MRI acquisitions. Additionally, machine learning methods are particularly useful for the study of multidimensional data sets. In this work, we have tested the reach of b-tensor encoding data analyses with machine learning in different histopathological scenarios. We achieved this in three steps: 1) We induced different levels of white matter damage in rodent optic nerves. 2) We obtained *ex vivo* DW-MRI data with b-tensor encoding schemes and calculated quantitative metrics using Q-space trajectory imaging. 3) We used a machine learning model to identify the main contributing features and built a voxel-wise probabilistic classification map of histological damage. Our results show that this model is sensitive to characteristics of microstructural damage. In conclusion, b-tensor encoded DW-MRI data analyzed with machine learning methods, have the potential to be further developed for the detection of histopathology and neurodegeneration.

## Introduction

Non-invasive inference of tissue microstructure is made possible through diffusion-weighted magnetic resonance imaging (DW-MRI) [1]. This valuable technique characterizes cerebral microstructure, connectivity, plasticity, development, and diverse pathologies. The need to find clinical standardized DW-MRI biomarkers in healthy and pathological neural tissue has driven more research in this field [2, 3]. Classical DW-MRI techniques (i.e., those encoding diffusion through a single pair of pulsed gradients) have shown sensitivity to nervous tissue damage but not specificity to diverse histopathological forms [3]. Multidimensional diffusion encoding (MDE) DW-MRI [4] techniques were developed to address this situation. Specifically, the b-tensor encoding technique [5] provides a solid framework to acquire multidimensional diffusion data. It has been used in controlled environments with simulations [6], healthy tissue [7], and in presence of pathology [8–10].

One of the main advantages of using b-tensor encoding acquisitions is that the complex information in the data is suitable for advanced diffusion models or signal representations. In the diffusion tensor distribution (DTD) model [4], a collection of micro-diffusion tensors with different shapes, sizes, and orientations describes microstructure. The Q-space trajectory imaging (QTI) method [5] extracts metrics from b-tensor encoding images to characterize the behavior of the DTD. Such complex micro-structural models are not attainable through standard DW-MRI acquisitions. Thus, MDE DW-MRI potentially describes neuropathological changes in detail. However, relatively few studies have used this technique for this purpose [11, 12].

In addition to novel DW-MRI acquisition methods, machine learning (ML) algorithms have revolutionized technological and scientific advancement in practically all fields. In biomedical imaging, there are multiple examples of ML applications like automatic image segmentation, data processing, MRI reconstruction, etc. In DW-MRI, ML techniques have been used for data pre-processing [13, 14], estimation of diffusion parameters [15–19], automatic white matter bundle segmentation [20], among other applications (for a review, see [21]). There are still, however, several opportunities for clinical applications and improvements in the detection of histopathology.

This work aims to evaluate the accuracy of ML for the classification of various degrees of white matter tissue damage based on metrics derived from QTI in an animal model. To this end, we performed experimental manipulations that induced histopathological changes in the optic nerve and obtained b-tensor encoding images *ex vivo*. Histological evaluation of the specimens provided the ground truth for a ML classifier, accounting for severity and spatial extent. We also identified the most relevant features used by the classifier. Finally, we show the model’s utility for detecting neurological damage on a probabilistic classification map.

## Materials and methods

### Animals

We used adult male Wistar rats for this study (weight: 354±59 g). Animals were held in a vivarium room under normal light/darkness conditions with controlled temperature and humidity. Animals had *ad libitum* access to food and water. The study was approved by the Bioethics Committee of the Institute of Neurobiology, Universidad Nacional Autónoma de México (protocol 096.A) under NOM-062-ZOO-1999 federal law. All procedures were performed in compliance with ARRIVE guidelines.

### Animal surgery

**Fig 1:**
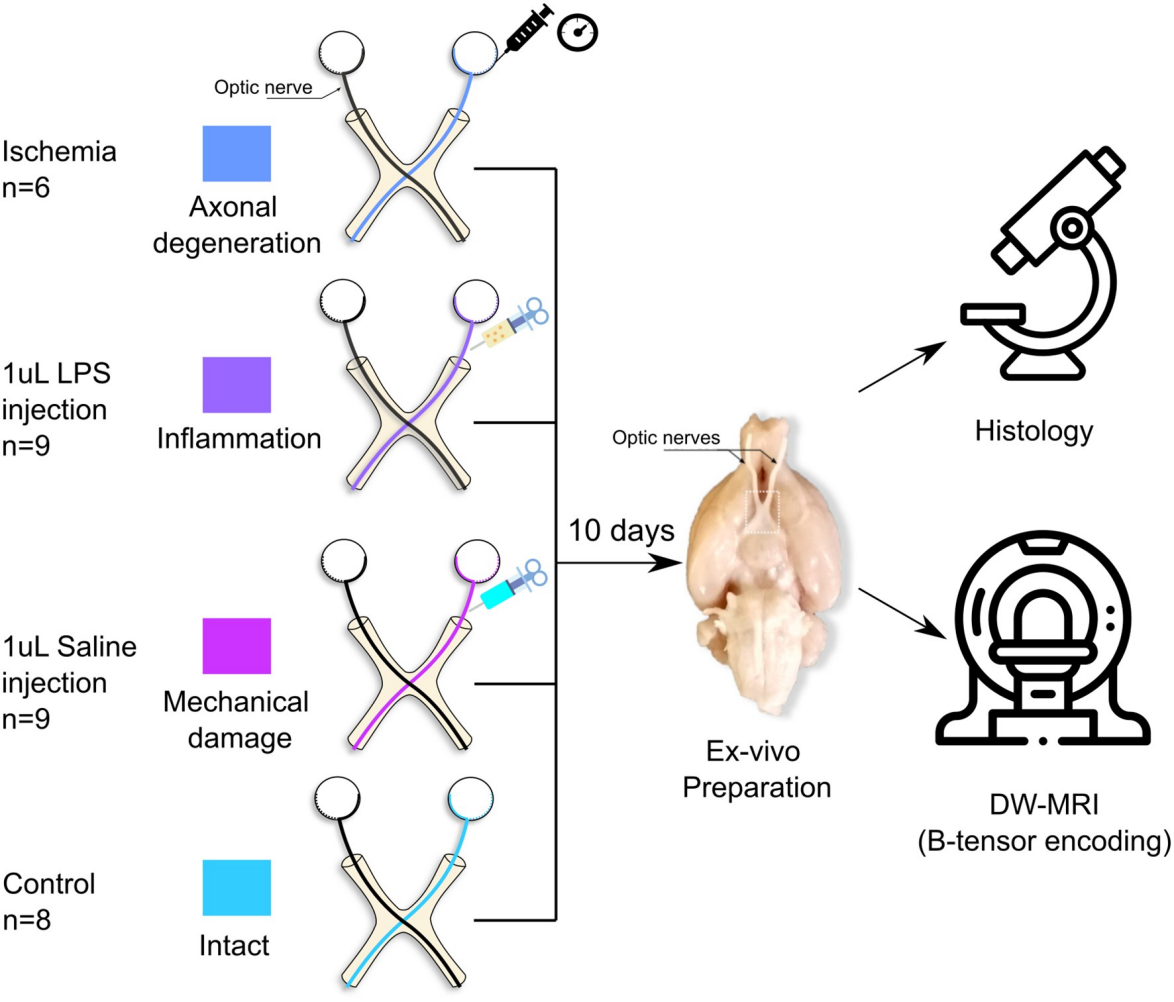
Experimental design. Axonal degeneration or inflammation of the right optic nerves was induced *in vivo* through retinal ischemia or LPS injection, respectively. Additionally, saline solution was injected into a group of animals to evaluate mechanical damage. Animals were sacrificed ten days after experimental procedures, tissue was fixed, and the brains and optic nerves were extracted. b-tensor encoding DW-MRI images were acquired *ex vivo*.

Normal rats were used to induce different forms of white matter pathology (Fig 1). Rats were anesthetized with a ketamine/xylazine mixture (70mg/kg and 10mg/kg ip) and placed on a well-illuminated surface. For each animal the right optic nerve was injured while the left one remained intact. This allows a direct comparison between subjects and between groups. Rats were divided into four different procedures as follows:

1. Axonal degeneration (n = 6). Induced through unilateral retinal ischemia [22, 23]. Animals were placed in a stereotaxic frame. A 32-gauge needle was inserted into the anterior chamber of the right eye of each rat, and connected to a reservoir with saline solution that was elevated until an in-line pressure monitor indicated 120 mmHg (higher than systolic pressure); this pressure was maintained for 90 min.
2. Inflammation (n = 9). Elicited through injection of 1*μl* of lipopolysaccharide (LPS, 4.5 *μg*/*μl*; Sigma-Aldrich) in the optic nerve [24]. A small lateral incision behind the eye was performed. Then, lacrimal glands and extra-ocular muscles were dissected to expose the optic nerve. Using a 32-gauge needle coupled to a Hamilton syringe, the injection was done approximately 1 cm rostral to the optic chiasm. After careful and slow manual injection, the needle was left in place for approximately 1 min to avoid reflux. The skin was sutured and topical antibiotics were administered. Animals were allowed to recover from anesthesia and placed in their cages until perfusion.
3. Saline solution injection (n = 9). This group was used to evaluate the mechanical damage produced solely by needle insertion. The procedure was identical to that of the previous group, but the injection consisted of 1 *μl* of saline solution.
4. Control (n = 8). Healthy animals with both optic nerves intact.

### Brain extraction

Ten days after the surgical procedure, all the animals were deeply anesthetized using an intraperitoneal overdose of sodium pentobarbital. Animals were transcardially perfused with 0.9% sodium chloride followed by paraformaldehyde (4%) glutaraldehyde (2.5%) solution. Brains were carefully extracted leaving at least 1 cm of optic nerves intact. Specimens were post-fixed in fresh 4% paraformaldehyde solution at 4°C until scanning day.

Following previous reports from our group (e.g. [23]), we did not rehydrate tissue prior to scanning. Recent work has shown that tissue rehydration increases T2 without altering the diffusion coefficient of white matter [25, 26]. While a longer T2 has the potential to increase the signal-to-noise ratio, our long acquisition protocol using a Helium-cooled coil (see below) produced high-quality images despite the lack of rehydration of tissue.

### Imaging

Brains were scanned 15±10 days post-extraction. The most distal portions of the optic nerves were attached to the ventral side of the olfactory bulbs by using cyanoacrylate to prevent the optic nerves from floating during the scan. To achieve a reduced field of view for DW-MRI, we carefully dissected and kept the basal portion of the brain. These specimens were immersed in Fluorinert (FC-40, Sigma-Aldrich) and allowed to rest for 4 h at room temperature before scanning. Acquisition protocols were carried out at the National Laboratory for Magnetic Resonance Imaging using a 7 T Bruker Pharmascan with 760 mT/m gradients and a Cryoprobe. The scanning room temperature was 21±1°C, and the Cryoprobe’s heated ceramic head mount was set at the same temperature. DW-MRI images were acquired using the available sequence in the Preclinical Neuro MRI repository (https://osf.io/ngu4a), which is based on a 2D spin-echo sequence with a single k-space line readout for each TR. Voxel resolution was 80 × 80 × 1000 *μm*^3^. Other MRI parameters include: TR = 1500 ms, TE = 30.9 ms, two averages, flip angle = 79°, scan time = 16 h.

DW-MRI images were obtained with b-tensor encoding based on a previously described protocol [7]; specific modifications were done for our *ex vivo* setting. The protocol consists of three different gradient waveforms to obtain linear, planar, and spherical tensor encodings (LTE, PTE, and STE, respectively). STE and PTE waveforms were optimized and Maxwell-compensated [27] using NOW toolbox [28]. LTE waveforms were extracted from the optimized STE waveforms to obtain similar gradient spectral characteristics between waveforms [29]. All waveforms had the same duration (*δ*_1_ = 9.8, *δ*_2_ = 10.4, separation time = 5.72 ms), and each one was scaled in gradient magnitude to achieve four different b-values (0.5, 1.4, 2.8 and 4 *ms*/*μm*^2^). The STE waveform was rotated to obtain 10 directions for every b-value. Rotating the STE waveforms results in the same spherical b-tensor, but this redundancy ensures the robustness of data processing [7]. LTE and PTE waveforms were rotated to obtain [10, 10, 16, 46] directions for each corresponding b-value. S1 Fig shows the waveforms and protocol used in this experiment.

### Image data preprocessing

The acquired images do not present many artifacts because of the long spin-echo based acquisition. Since the high b-value shells (4 *ms*/*μm*^2^) are noisy, the only preprocessing step needed was denoising, which we achieved through Marčenko-Pastur principal component analysis [30, 31] as implemented in *mrtrix3* (version = 3.0.0) [32]. Examples of final images for each encoding acquisition are shown in S2 Fig. Regions of interest (ROIs) for experimental and control optic nerves were manually drawn in 3 to 4 slices per nerve (92 ± 25 voxels for each nerve).

### Analysis of b-tensor encoded DW-MRI

We used QTI analysis to extract eight microstructural measures from the obtained b-tensor encoding images. Four of them capture the macroscopic behavior of the DTD ensemble and are akin to those from diffusion tensor imaging (DTI) [33]: 1. Fractional Anisotropy (FA). 2. Mean diffusivity (MD). 3. Axial diffusivity (AD). 4. Radial diffusivity (RD).

The following four QTI metrics capture the microscopic behavior of the DTD ensemble and are only achievable through methods such as b-tensor encoding:

1. Micro fractional anisotropy (*μ*FA). Measures the average microscopic anisotropy of all tensors in the DTD.
2. Orientation coherence (*C*_*c*_). Measures the level of orientation coherence of the micro tensors in the DTD.
3. Isotropic kurtosis (*K*_*i*_). Quantifies the kurtosis produced by the size variance of the micro tensors in the DTD.
4. Anisotropic kurtosis (*K*_*a*_). Quantifies the kurtosis produced by the microscopic anisotropy.

We obtained QTI metrics using the implementation in QTI+ [34]. Standard QTI implementation is biased to complex microstructure [6], whereas QTI+ provides a more stable solution to the DTD fitting optimization problem and achieves smoother and more precise maps than QTI [34]. We used the default settings for QTI+ [35]. To avoid regions where QTI fitting was poor, we excluded voxels (6.8% of all data) where any of the QTI metrics resulted in values outside their valid range: Normalized metrics (FA, *μ*FA) should lie between 0 and 1, and kurtosis metrics (*K*_*i*_ and *K*_*a*_) should be between 0 and 5.

### Histology

Following DW-MRI acquisition, specimens were returned to 4% paraformaldehyde solution and kept at 4°C until processing. Briefly, the optic nerves were separated from the basal portion of the brain and were washed with buffered sodium cacodylate (0.1 M) and glutaraldehyde (3%). Then, they were stained with osmium tetroxide (0.1%), washed with cacodylate buffer (0.1 M), and dehydrated with ethyl alcohol at different concentrations (10%, 20%, 30%, until absolute). Next, samples were embedded in a 1:1 epoxy resin/propylene oxide solution for 12 h. For polymerization, samples were placed in a plastic container with epoxy resin and kept at 60°C for 36 h. Finally, each block was sectioned (600 nm thick) using an ultramicrotome (RMC PowerTome PT XL). Slices were stained with a toluidine blue/sodium tetraborate solution (both 5%). Toluidine blue stains a wide variety of nuclear, cytoplasmic, and intercellular matrix structures [36, 37]. Because of its polychromatic nature, nuclei and ribosomes are both stained deep blue, while membranous structures are stained blue-green. Other components are stained deep blue (e.g. collagen) or pale green-to blue (e.g. basal laminae). The addition of osmium tetroxide provides clear staining of membranes and lipid-rich myelin sheaths [38, 39]

Photomicrographs were obtained with a Leica DM750 microscope (equipped with a 5M pixels digital camera) with 10x and 100x objectives, and an Amscope T690C-PL microscope (equipped with a 10M pixels digital camera) with a 40x objective. We transformed the images to 16-bit grayscale and digitally enhanced their contrast using Fiji [40] (version = 2.9.0). Images with the 40x lens were stitched using the stitching plug-in [41] available in Fiji.

### Machine learning pipeline

**Fig 2:**
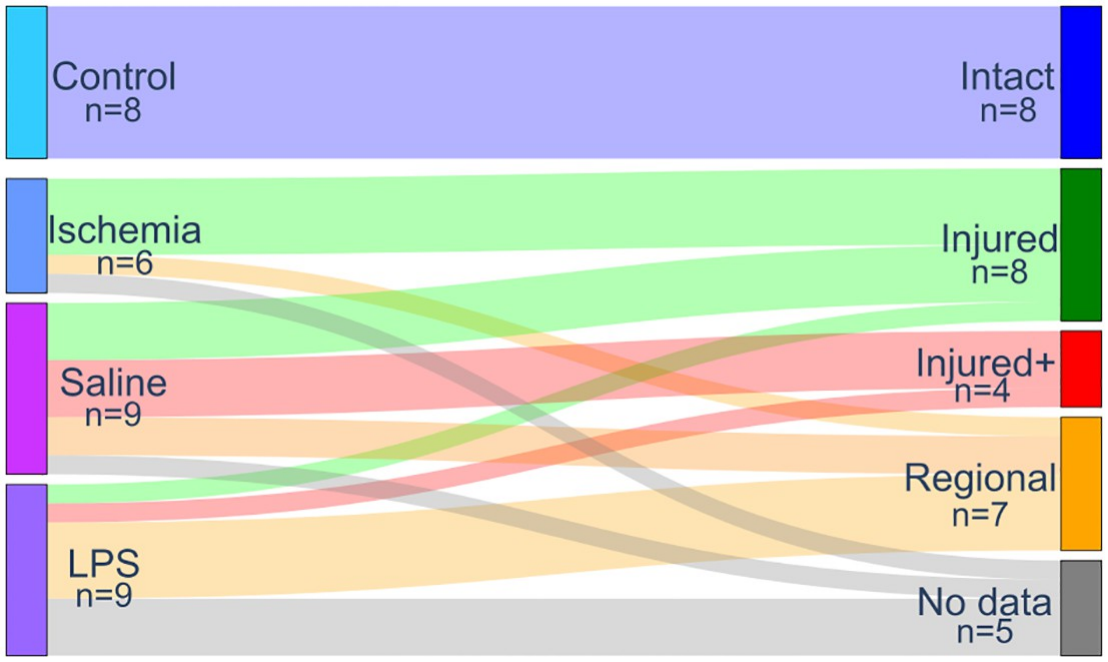
Labeling system based on histological patterns. Classes were assigned to each nerve after visual examination of histology, based on the spatial pattern and type of histological characteristics. The left column represents the experimental procedures, while the right column indicates the labels used for the identification of tissue types based on diffusion properties. There were five specimens without histological data available (not included in analyses). Line thickness represents the proportion of nerves mapping from experimental to histological labels.

Visual inspection of the photomicrographs revealed histological patterns that overlapped between experimental groups (see Histological evaluation). Histopathological features secondary to inflammation and degeneration were compounded by mechanical damage caused by the injection of either saline solution or LPS. We therefore chose to (*i*) reformulate the classification labels for the ML pipeline into histological classes that reflect different types of histopathological damage (Intact, Injured, and Injured+) and (*ii*) analyze voxels of the regionally affected nerves, identified as the Regional pattern (see Fig 2). The number of voxels included in this analysis was 821, 561, 374, and 731 (Intact, Injured, Injured+, and Regional, respectively).

**Fig 3:**
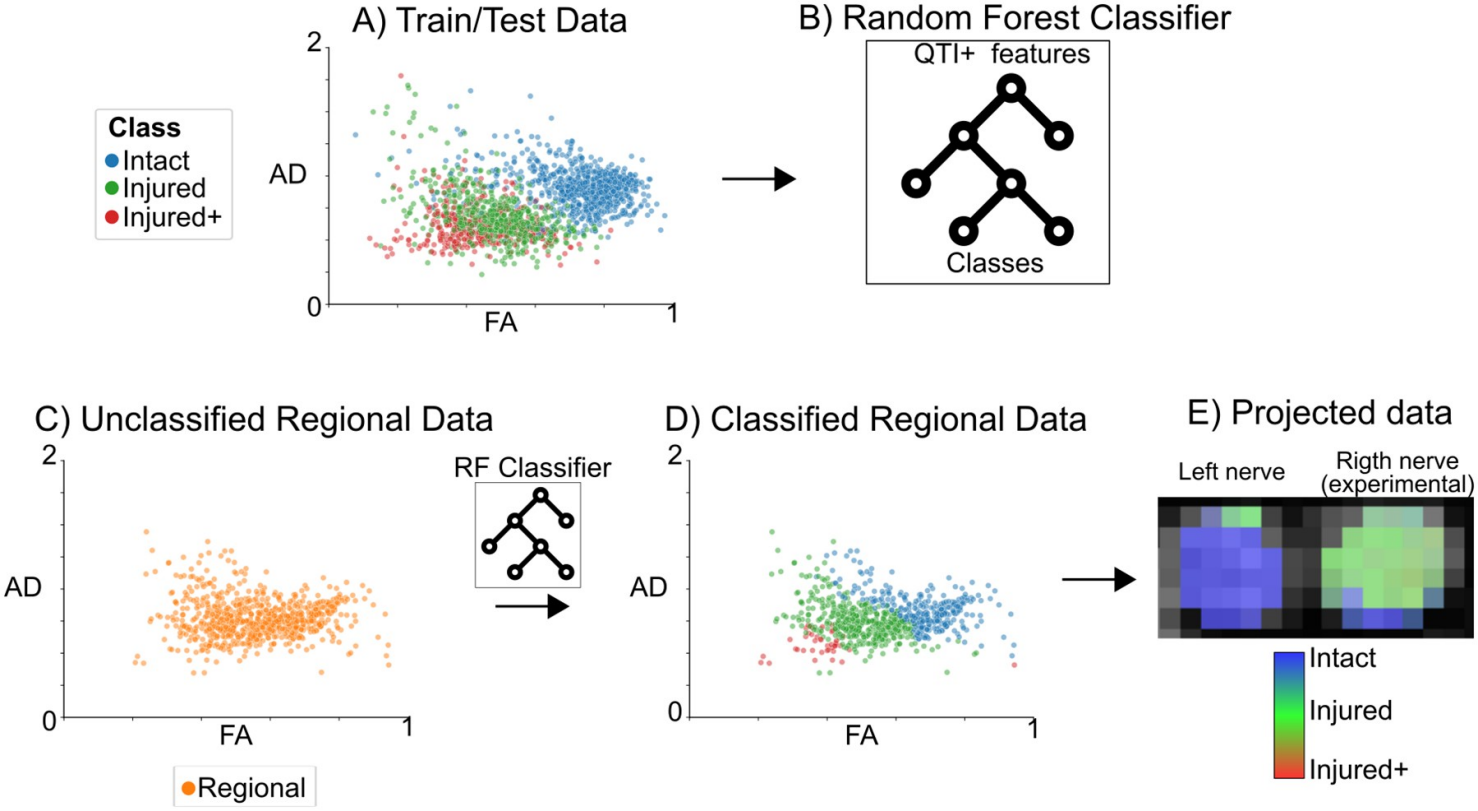
Diagram of the machine learning pipeline. We used the QTI+ data for all voxels labeled according to histology (Panel A: each color-coded data point represents a voxel) as input to train/test the random forest model (B). We classified each voxel of the regionally-affected nerves (Regional) (C) into histological damage classes (D). Finally, we projected the classified data back into an anatomic RGB map that quantifies tissue damage (E).

Fig 3 shows a diagram with the ML pipeline. QTI+ data from Intact, Injured, and Injured+ classes were used for the train/test set in the ML pipeline in a voxel-wise fashion (A). We trained a random forest model [42] (B) (80/20% fold) and conducted a feature relevance analysis with Gini importance [42] using scikit-learn (version = 1.1.2, https://scikit-learn.org). We tuned the hyperparameters for the random forest model using the grid search method in scikit-learn with cross-validation (*k* = 4) enabled. The optimized hyperparameters are: number of estimators [trees]=100, maximum depth = 6, splitting criteria = Gini impurity, minimum number of samples required to be at a leaf node = 2, minimum number of samples required to split an internal node = 7. The rest of the hyperparameters have the default values defined in scikit-learn (version = 1.1.2). We classified each voxel in the Regional nerves with this model (C and D). The resulting probability of class membership is visualized as a composite red-green-blue (RGB) map (E), with each channel representing a tissue class: Intact:Blue, Injured:Green, and Injured+:Red.

Feature relevance analysis is a complex subject with potential caveats. Previous work indicates that Gini importance has two main problems: First, it tends to be biased towards features with high cardinality [43]. This, however, does not apply to our data because they are on a continuum. Second, Gini importance reports statistics related to the training set [42]. Thus, we also performed a feature relevance analysis by permutations on the test set [42]. After we reported the accuracy/F1-Score results and feature analysis with the test set, we calculated a bootstrapped estimator to determine the variance of the permutation feature analysis and checked if the order of relevance in our results remained the same. To this end, we randomly permuted the train/test partitions to perform 200 different experiments (using the same optimized hyperparameters reported for the random forest model) to evaluate the reproducibility of the permutation feature relevance analysis. We emphasize that this analysis was done after the main analysis with the train/test set that is reported in the Machine learning pipeline (Machine learning classification), and its only purpose is to check feature analysis biases related to the original train/test partition.

## Results

### Experimental labels for DW-MRI data

**Fig 4:**
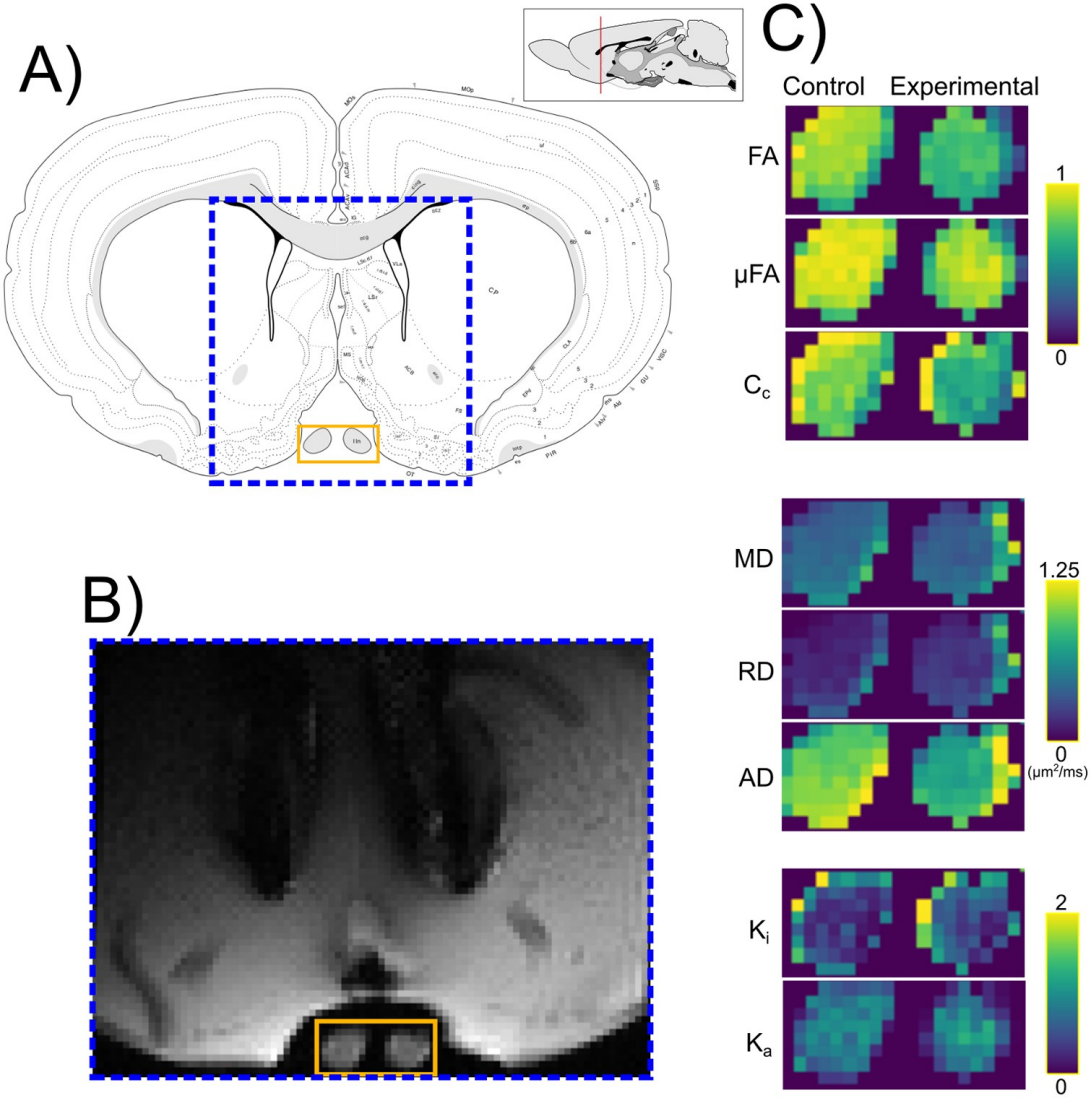
Q-space trajectory imaging contrasts. A) Anatomical atlas reference (adapted from [44]). DW-MRI images were obtained from the portion of the brain specimen indicated by the dashed blue box. B) Example of denoised DW-MRI image with spherical b-tensor encoding (b = 2.8 *ms*/*μm*^2^) from the Ischemic group. C) Enlarged images corresponding to the orange rectangle in panel B. QTI metrics for control (left) and experimental (right) optic nerves. Abbreviations: fractional anisotropy (FA), microscopic fractional anisotropy (*μ*FA), orientation coherence (*C*_*c*_), mean diffusivity (MD), radial diffusivity (RD), axial diffusivity (AD), isotropic kurtosis (*K*_*i*_) and anisotropic kurtosis (*K*_*a*_).

**Fig 5:**
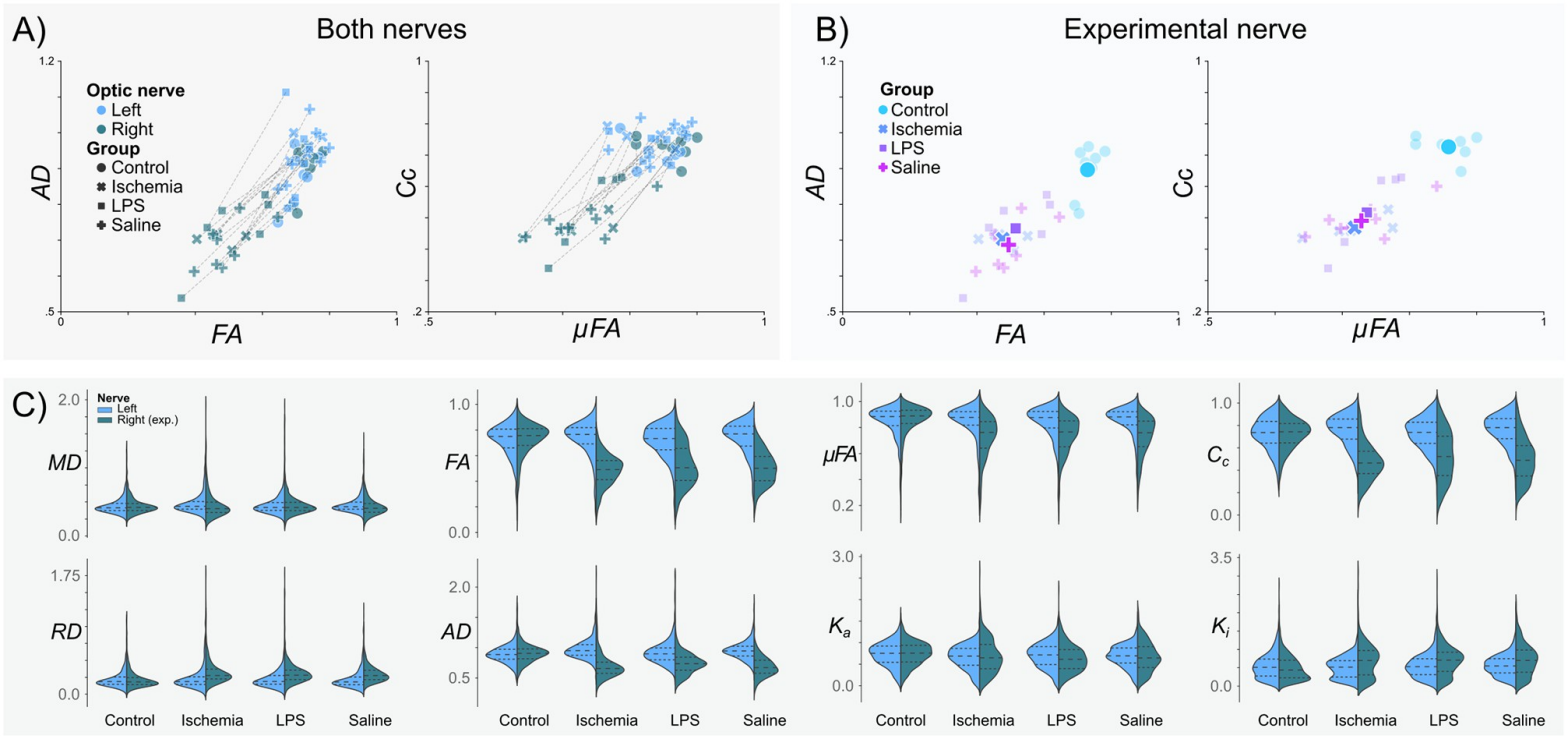
Q-space trajectory imaging metrics by experimental group. A) Intact (Left) vs Experimental (Right) optic nerves. Data points correspond to the average values of all voxels of each optic nerve, per subject. Lines connect the two optic nerves of each subject. B) Right (experimental) optic nerves’ metrics color-coded according to the experimental procedure. Semi-transparent markers show average values per animal; average values for each experimental condition are indicated as large solid markers. C) Violin plots for the different experimental groups for each QTI metric.

Quantitative maps derived from QTI+ showed asymmetry between the intact and experimental nerves for most metrics (Fig 4). Fig 5A shows the per-animal average difference between the intact (left) and experimental nerve (right), indicating considerable differences between the two nerves. However, diffusion metrics from nerves in the experimental conditions (Fig 5B) showed a noticeable overlap. Fig 5C presents the overall diffusion metrics distribution for all voxels by experimental groups.

### Histological evaluation

**Fig 6:**
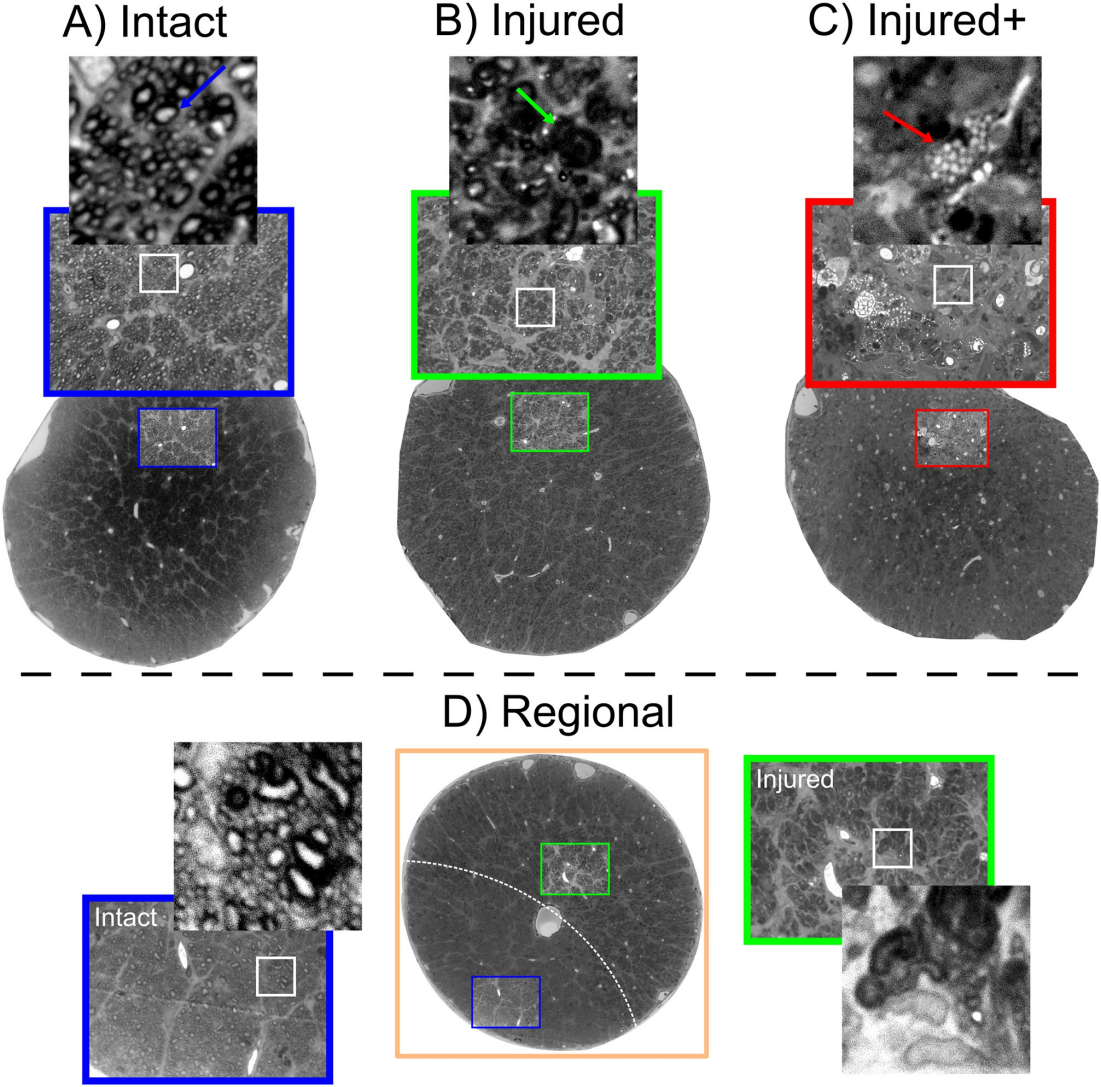
Histopathological patterns after experimental procedures. A: Intact nerve with a large number of axons and narrow glial processes. Axons show bright axoplasm and dark surrounding myelin sheaths (blue arrow). B: Injured nerve with collapsed axons (green arrow), reduced number of viable axons, and gliosis. C: Injured+ nerve with very few axons and large reactive glial processes with foamy interior indicative of myelin degradation (red arrow). D: Regional nerve showing clearly separated areas (dashed white line) of either of the three histological patterns. The areas in the Regional nerves have characteristics of the Intact, Injured, and Injured+ classes, making them a suitable fit for a machine-learning classification problem. Photomicrographs of whole nerves acquired at 10x magnification; photomicrographs in colored squares acquired at 100x magnification.

Histological examination of sections stained with toluidine blue (see Histology) showed that retinal ischemia induced diffuse axonal degeneration and mild gliosis. Nerves injected with LPS also had reductions of axonal density and more glial cell infiltration. There was evidence of independent mechanical damage, as saline solution injections led to axonal degeneration and gliosis ranging from mild to severe. Moreover, while retinal ischemia induced tissue injury mostly in a spatially homogeneous fashion, nerves treated with either type of injection produced damage either homogeneously or only within confined regions of the nerve, with some areas showing damage and others displaying a nearly intact structure. Thus, we manually labeled each nerve based on the type and spatial pattern of histopathology, as (1) *Intact*; (2) *Injured*: characterized by globally reduced axonal density; (3) *Injured+*: displaying homogeneous and profound axonal loss and severe infiltration of glial cells and macrophages with foamy appearance; and (4) *Regional*, with different regions of the nerves showing one of the three histological types (Fig 2). There were five specimens with no histological data available and were therefore excluded from all analyses. This classification system allowed us to perform a spatial assessment of microstructural damage produced by the experiments (i.e., we used the diffusion properties of the Intact, Injured, and Injured+ classes to identify the corresponding histological patterns in the regionally affected nerves). Photomicrographs in Fig 6 show examples of the histopathological patterns identified. Panel A is a prototypical *Intact* nerve, characterized by a large number of axons with clearly-defined myelin sheaths and bright intra-axonal space, interspersed with angular glial cell processes. Panel B shows an *Injured* nerve, displaying reduced axonal density, numerous collapsed axons with dark intra-axonal space (green arrow), and reactive glial cells with large, amoeboid processes. Panel C shows an *Injured+* nerve, nearly devoid of axons with a considerable amount of glia in a reactive foamy state (red arrow). S3 Fig provides further examples of these histopathological characteristics. Lastly, panel D shows a Regional nerve, with large and clearly delimited regions that can be described with the three aforementioned classes. As noted in Figs 2 and 3, this histological-type classification was used to perform a voxel-wise ML-based classification from QTI+ metrics.

### DW-MRI metrics based on histological labels

**Fig 7:**
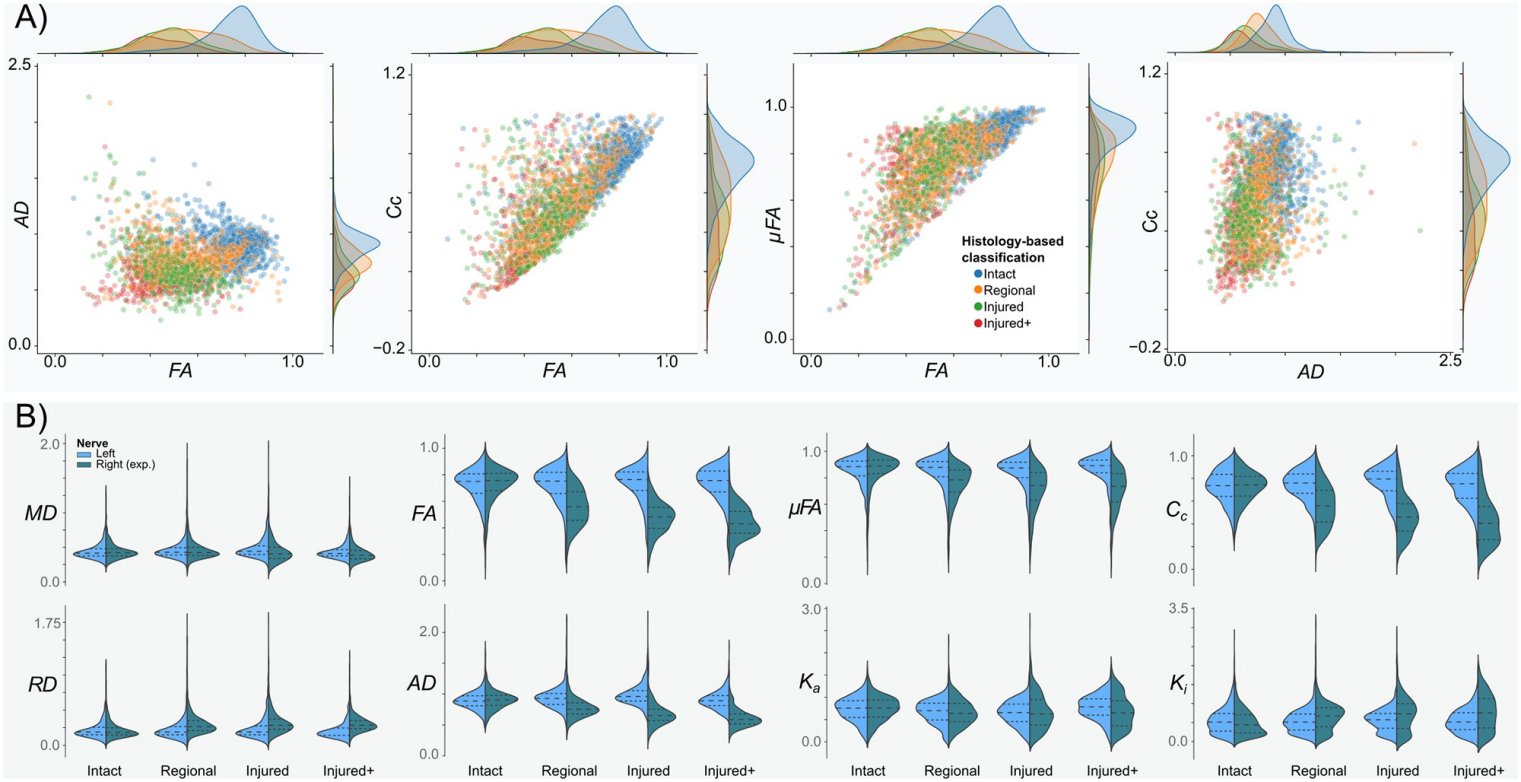
Q-space trajectory imaging metrics by the histology-based label. A) Voxel-wise scatter plots according to the histology-based labeling system. Metrics from the Intact class (blue) are clearly different from those of the experimental classes. Metrics from Injured and Injured+ classes are overlapped but still separable. The Regional class, being composed of areas of either Intact or any of the two injured classes, shows diffusion metrics distributed across the metrics space. B) Violin plots for the histology-based labels for each QTI metric.

A voxel-wise inspection according to the histology-based classes of the right (experimental) nerves revealed differences in the distributions for QTI metrics in groups (Fig 7A). Albeit their large overlap, it is possible to visually separate the distributions. Group-wise analyses (Fig 7B) showed considerable alterations of QTI metrics in all histopathological types, characterized by reduced FA, AD, *μ*FA, and *C*_*c*_, and increased radial diffusivity and isotropic kurtosis (*K*_*i*_). Mean diffusivity showed slight reductions in the Injured and Injured+ conditions.

### Machine learning classification

**Fig 8:**
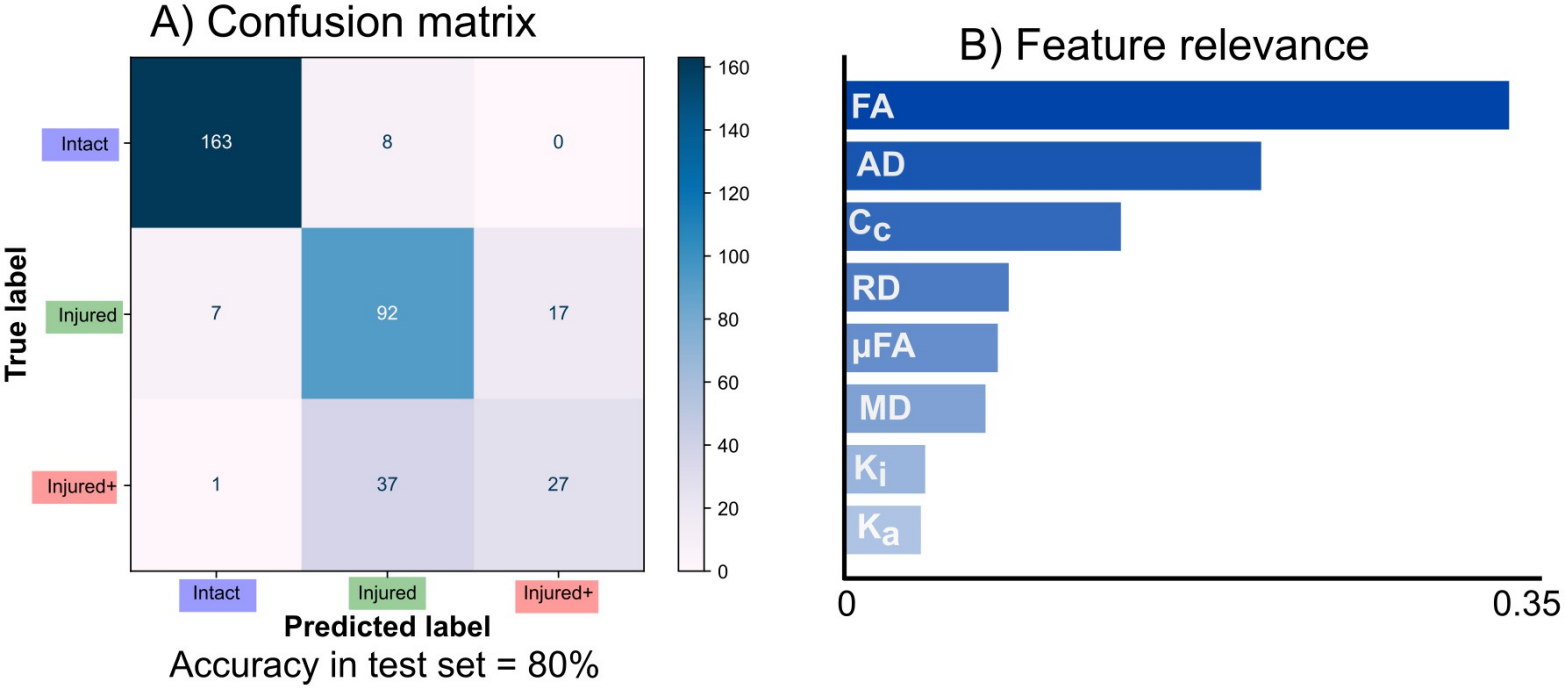
Machine learning results. A: Confusion matrix for classification in the test set. B: Feature relevance from the random forest. QTI-derived FA and AD (which can also be derived from DTI), are highly important for classification. With the exception of *C*_*c*_, metrics exclusively derived from QTI are less relevant.

We trained a random forest model for the voxel-wise classification of histopathological classes in nerves identified as having Regional abnormalities, according to the pipeline in Fig 3. The overall classification accuracy was 80.11% and an F1-score of 79.4% (with a weighted average for multiclass classification) to distinguish between the three histopathological classes. Fig 8A shows the confusion matrix for the classification of the test data set. Fig 8B shows the results of the feature relevance analysis. FA and AD (derived from QTI in this work, but also possible to calculate with DTI) are the two most relevant features for the ML model. S4 Fig shows the permutation feature analysis and the bootstrapped feature analyses, which confirmed the relevance of FA, AD and *C*_*c*_ for classification, in that order.

In addition to illustrating the classification pipeline, Fig 3D shows voxels from Regional nerves classified with the ML method. Fig 3E shows an example of classified voxels as an RGB map. The majority of voxels within left nerves (Intact) are correctly classified (blue–Intact). The right (experimental) nerves show most of the voxels identified as Injured, with spatial patterns that correspond to histology (see Fig 6).

**Fig 9:**
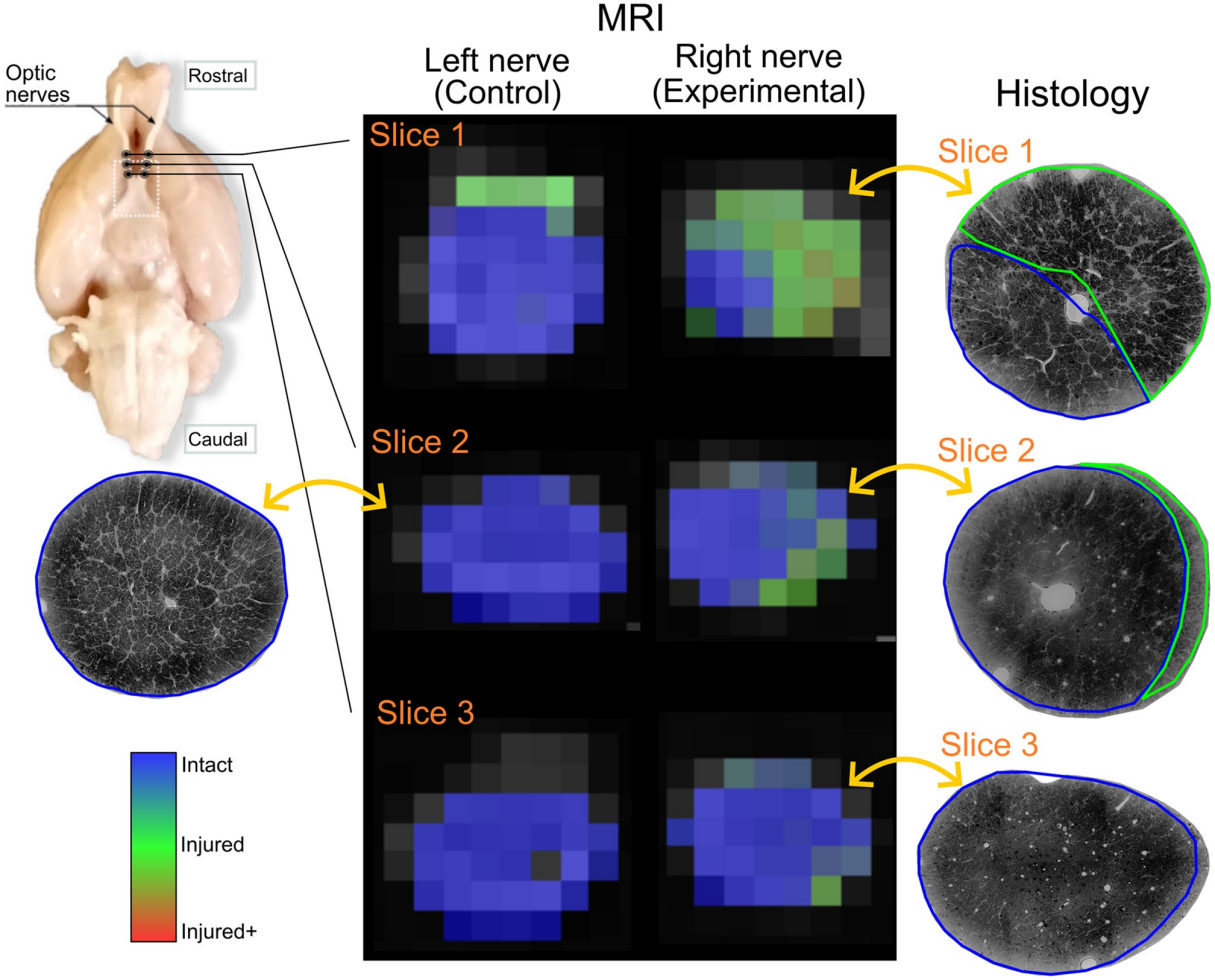
Voxel-wise classification of histological patterns. Rat histological example data showing Regional damage of the left and right (experimental) nerves. The two optic nerves are shown in three different slices in rostro-caudal order. Photomicrographs of the same experimental nerve at approximately the same locations show clearly demarcated areas of Injured and Intact histological patterns, that correspond to the voxel-wise classification of the DW-MRI of the experimental nerve. The few voxels incorrectly classified as Injured in the left (control) nerve likely result from partial volume effects.

An example voxel-wise classification of a single nerve (LPS injection and identified as Regional, see Fig 2) shows the algorithm is sensitive to microstructural degeneration (Fig 9). Voxels identified as Injured and Injured+ are larger in number in rostral slices (i.e., nearest to the injection site), with more caudal slices gradually showing more voxels classified as Intact. Notably, photomicrographs of the same nerve at approximately the same levels as the DW-MRI exhibit similar spatial patterns of injury and corresponding histopathological classes as identified by the random forest. The vast majority of voxels in the left (intact) nerves are correctly identified as Intact. S5 Fig shows three more examples of correct histopathological damage classification.

## Discussion

In this work, we explore the synergy of QTI metrics and ML for the non-invasive identification of white matter histological damage. Our data show that these metrics are sensitive to altered histological patterns. Three metrics (two that can also be derived using DTI and one exclusive to multidimensional encoding methods) were the most relevant for the accurate classification of tissue damage. Notably, the metric achievable only through b-tensor encoding further improved the results obtained from the ML pipeline.

The optic nerve has been widely used to evaluate white matter changes through DW-MRI. A common approach is to induce retinal ischemia that results in Wallerian degeneration of the retinal ganglion cells and their axons throughout the lesioned nerve, which reflects as specific patterns of diffusion abnormalities [23, 45]. Differentiation between inflammation and axonal degeneration through DW-MRI is an active topic of research. However, we did not observe major differences between optic nerves mechanically damaged by the injection of saline solution, and those injected with LPS with the intent to induce inflammation, as both showed axonal degeneration and gliosis of various degrees of severity. For this reason, this study focused on examining the levels of severity of histopathology using ML methods, with the aim to improve the diagnostic yield of DW-MRI.

Injections of the nerve produced enough tissue damage to reduce FA, similar to the reductions caused by retinal ischemia (Fig 5) [23, 45]. Decreased *μ*FA was also observed in all experimental conditions, indicative of an increase in isotropic diffusion profiles from the axonal loss produced by retinal ischemia and tissue damage induced by injection of optic nerves (Fig 6). This observation agrees with previous literature that suggests axonal loss decreases *μ*FA [5, 8, 46]. Coherence, as seen with *C*_*c*_, was also reduced in all affected nerves, which fits the observed tissue disorganization of the experimental nerves. We hypothesize that in addition to the loss of coherence, infiltration of glial cells may further reduce both indices of anisotropy. Overall, the metrics derived from QTI overlapped among the experimental conditions (Fig 5B). This prevented us from establishing a clear differentiation between inflammation and axonal degeneration. However, as discussed in Histological evaluation, our experimental groups shared histopathological characteristics with overlapping diffusion profiles. For this reason, we cannot conclude whether QTI is capable of differentiating both pathological events.

We observed that mechanical damage varied from subtle axonal degeneration to the annihilation of the entire axonal population (Fig 6). We therefore re-classified our data based on histological findings and their spatial extent (Fig 2), with the Injured nerves (characterized by mild axonal population loss) and Injured+ (distinguished by strong axonal population loss and foamy reactive glia that usually appear only in advanced stages of degeneration [47–49]). In addition, many injected nerves showed a mosaic of Intact, Injured, and Injured+ histopathologies, which we set out to automatically classify based on the diffusion profiles derived from nerves with spatially homogeneous tissue characteristics. As we were working with complex eight-dimensional data from thousands of voxels, this was an ideal setting for an ML application.

Random forest models were selected because 1) they are less prone to overfit; 2) they are easier to interpret (individual estimators–i.e., decision trees–in the model can be inspected and interpreted); 3) the variance in the estimators provides resilience to (*i*) noise and (*ii*) poor quality data points; and 4) feature relevance analysis is straightforward. We obtained similar accuracy results when using other state-of-the-art ML methods like XGBoost [50] (accuracy = 80.38%) and neural networks [51] (accuracy = 80.68%). This indicates that classification performance is more related to the nature of our data than to the classification algorithm used.

The overall accuracy performance of the automatic classification using random forest was high (80%). While the best distinction performance was between Intact and the two Injured classes, there was a modest success in the differentiation between the Injured and Injured+ classes (Fig 8A). Confusion between the two degrees of injury may be due to axonal loss (present in both types) acting as the main microstructural characteristic driving the measured diffusion properties. Other DW-MRI modalities specific to glial cells [52] or combined with other MRI modalities like spectroscopy [53] could explain these cases.

Feature relevance analysis (Fig 8B) revealed that FA and AD are the most relevant diffusion metrics to differentiate between tissue types. This was expected, as both are sensitive to the overall loss of anisotropy in white matter capturing the main effect of degeneration. In regions of white matter with coherent axons (such as the optic nerves), FA can be reduced by the loss of microscopic anisotropy, reduced coherence of these microenvironments, or both. Hence, DTI metrics are sensitive but not specific. We expected features exclusive to b-tensor encoding to improve the classification algorithm by providing additional information, given their specificity to certain properties of microstructure [11]. In this light, *C*_*c*_ should capture the increased axonal dispersion characteristic of white matter degeneration [54], while *μ*FA would inform of the reduction of microscopic anisotropy. Indeed, *C*_*c*_ was the third most important feature in the analysis. Nevertheless, *C*_*c*_ is correlated to FA (*C*_*c*_ by QTI definition is the ratio of FA² to *μ*FA²) and therefore contributes less to the classification problem if FA is already included in the analysis. Repeating the same pipeline using only QTI features revealed that *C*_*c*_ is the most relevant feature of the analysis, followed by *μ*FA, while preserving a similar classification performance (not shown). We hypothesize that gliosis reduces *μ*FA in a similar pattern in all experiments, thus decreasing its efficacy as a predictor. We had also expected *K*_*i*_ (related to the variance of sizes in the DTD model [5]) to increase as a result of glial infiltration. *K*_*a*_ might explain the loss of micro anisotropy in the medium and is also related to axonal loss. The relatively low explained variance in the data by the kurtosis metrics may be attributed to the bias secondary to the assumption of the DTD model that *μK* is equal to zero [55], which is not the case in degeneration [56], and therefore *K*_*a*_ and *K*_*i*_ may both be absorbing this effect. In this work using a ML algorithm trained with data from white matter with a single coherently-aligned axonal population, features achievable with DTI (FA and AD) captured the main properties of neurodegeneration relevant to the classification. However, orientation coherence (*C*_*c*_) and *μ*FA could be important factors for the detection and staging of neurodegeneration in white matter regions with crossing fibers or in gray matter, where FA is confounded by the complexity of tissue architecture.

There are some limitations in this study. First, the experimental procedures (particularly those related to direct nerve injections) produced overlapping histopathologies. This precluded the distinction between axonal degeneration and inflammation, and limited the interpretability of our findings. In particular, we cannot conclude from our data whether QTI is capable of resolving between those two histopathological processes. However, careful examination of histological slides allowed us to differentiate between Injured and Injured+ classes based on the presence of foamy glial cells and the extent of axonal loss, which were identified by the random forest algorithm based on diffusion metrics. Future work should try to minimize confounding factors introduced by mechanical damage of the tissue by utilizing other experimental approaches. Second, other histological methods can improve the distinction between different histopathological processes. We used toluidine blue for its ability to provide accurate morphological details, as seen in our previous work [23]. Nevertheless, tissue preparation for this stain is incompatible with other histological methods, such as immunohistochemistry or immunofluorescence of glial cells, that could give additional information for histopathology classification. Third, the slice thickness of DW-MRI was large (1 mm). Thick slices were acquired to improve the signal-to-noise ratio, but partial volume effects could introduce inaccuracies in the estimation of diffusion metrics, particularly for the Regional pattern as injured regions vary along the nerve. Fourth, STE and LTE waveforms were tuned [7, 29], but this does not ensure they have the same diffusion time window [57]. Diffusion time dependence could be an important factor in neurodegeneration [3] and was not directly investigated or controlled for in this study; further studies should give some insight into the contribution of time-dependent diffusion to distinguish between types of histological damage. Last, ML applications benefit from large data sets. While our voxel-wise data set is not small, the overall accuracy of the method could be improved with more data points.

There are other possibilities for the analysis of b-tensor encoding data. Like the diffusion tensor, QTI is a signal representation [58]. There are other interesting avenues of analysis like DTD imaging [59] that can extract direct DTD features or even extend it to multidimensional MRI analysis to capture relaxometry effects [60, 61]. Approaches with biophysical models using b-tensor encoding [62, 63] can be used to extract microstructural properties that cannot be obtained without strong modeling assumptions using single diffusion encoding acquisitions. Nevertheless, they are based on the standard model of white matter that is applicable for healthy tissue, and it is unknown whether they would be adequate for the detection of severe deviations (i.e., tissue damage) without modifications to the underlying assumptions. More work is needed to test if these approaches to DW-MRI could identify tissue damage with high sensitivity and specificity.

Machine learning methods provide a new paradigm to understand and use the advanced methods available in the DW-MRI field. Although it serves as a proof of concept, the visualization of tissue type probabilities as a color map offers a straightforward means of qualitatively assessing the level of damage present at each voxel (Fig 9). The combination of spatial specificity and the availability of quantitative diffusion metrics can be a powerful tool to evaluate and diagnose microstructural changes in neurological disorders.

## Conclusion

In this work, we explored the ability of b-tensor encoding methods to detect and differentiate between different levels of white matter degeneration. Specifically, we explored the metrics derived from QTI using state-of-the-art machine learning methods. The majority of QTI metrics are sensitive to microstructural changes induced by neuropathology. While classic DTI metrics were the most important features for the training phase in the machine learning algorithm, features exclusive to b-tensor encoding improved its precision.

## Supporting information

S1 Fig Protocol scheme. Full protocol scheme and example waveforms (b = 2.8 *ms*/*μm*^2^) used in this study.
(TIF)

S2 Fig b-tensor encoding example images. Example of preprocessed DW-MRI acquired by b-tensor encoding (b = 2.8 *ms*/*μm*^2^) of a single slice from one representative animal in the retinal ischemia group. Linear, planar and spherical tensor encodings (LTE, PTE, STE) and a non-diffusion-weighted image (b = 0 *ms*/*μm*^2^) are shown. The yellow rectangle indicates the optic nerves.
(TIF)

S3 Fig Photomicrograph examples of histological classes. Photomicrograph samples of nine different specimens in the study. Columns share unique histological characteristics that represent each class. Intact: Densely-packed myelinated axons (blue circles), and normally appearing sharp, elongated glial processes (blue squares). Injured: Multiple collapsed axons (green circles) interspersed with small viable axons (small blue circles), surrounded by enlarged ameboid glial processes (green squares). Injured+: There is no axonal population left, with abundant large ameboid glial processes (green squares), many of which have foamy interiors (red squares). Purple asterisks correspond to blood vessels.
(TIF)

S4 Fig Feature relevance analysis. A) Permutation feature relevance analysis in the test set. B) Bootstrapped permutation feature analysis. FA and AD are the most important features. Gini importance (B) showed *C*_*c*_ as the third-ranking relevant feature.
(TIF)

S5 Fig Examples of regional histological damage and corresponding machine learning classification based on MDE DW-MRI. (TIF)
