# Supplementary figures and images for: Differentiation of white matter histopathology using b-tensor encoding and machine learning
Source: PLoS One. 2023 Jun 23;18(6):e0282549. doi: 10.1371/journal.pone.0282549 (PMC10289327; doi:10.1371/journal.pone.0282549)

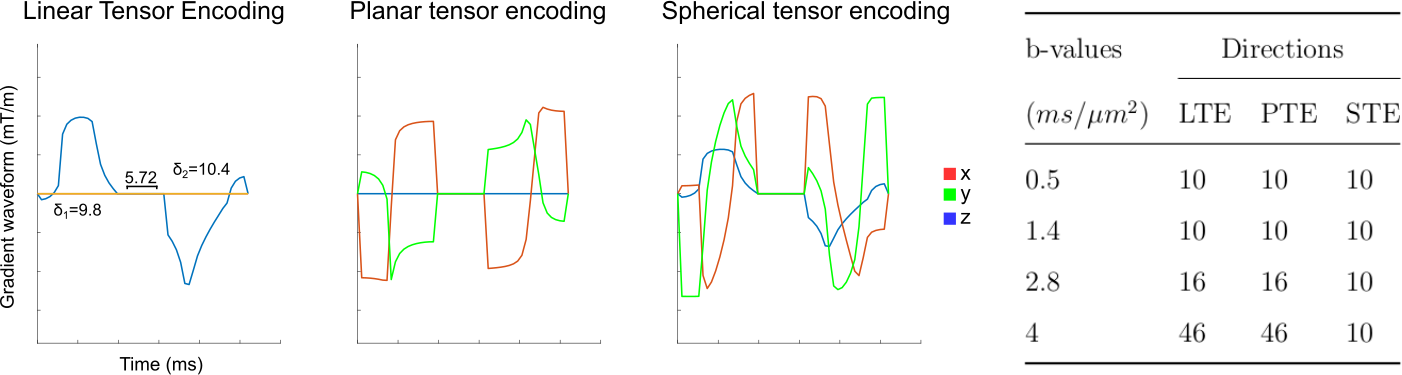

Supplement: S1 Fig — Full protocol scheme and example waveforms (b = 2.8 ms/μm2) used in this study. (TIF) [file pone.0282549.s001.tif]

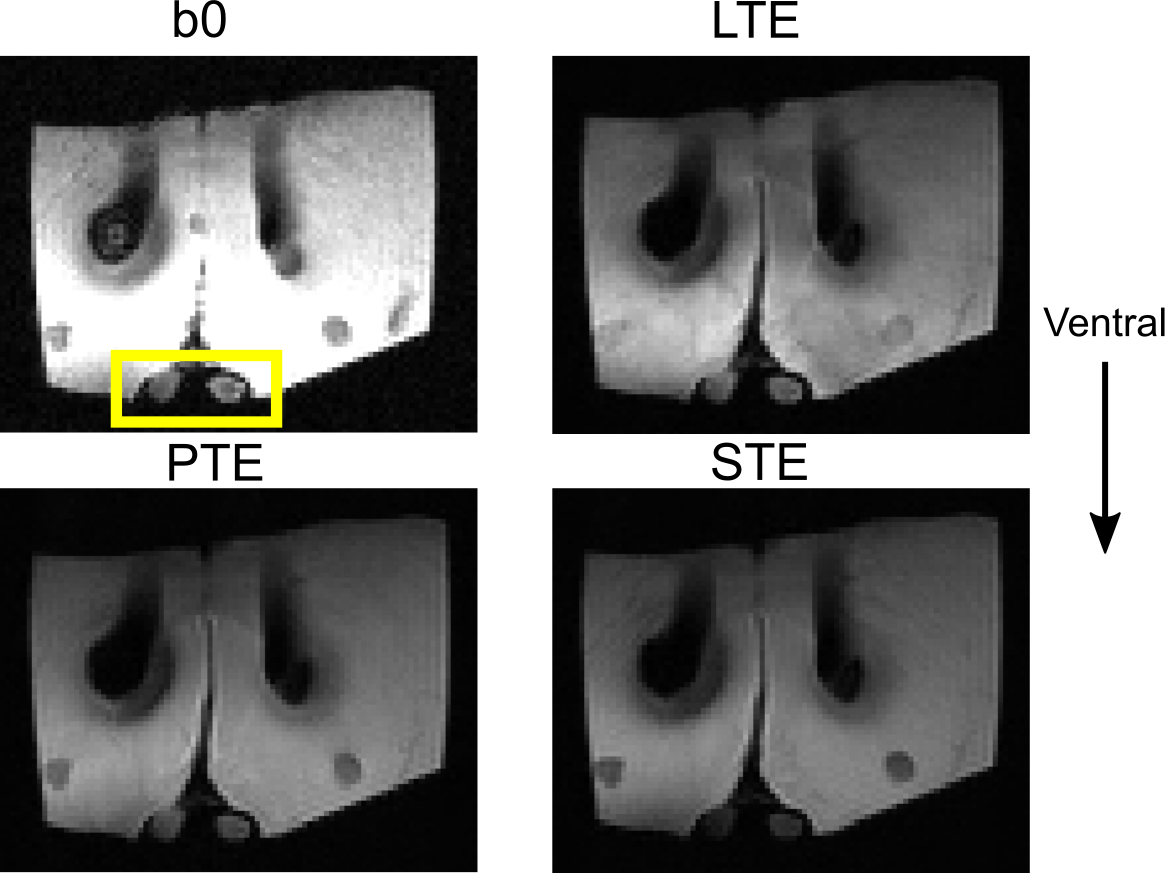

Supplement: S2 Fig — Example of preprocessed DW-MRI acquired by b-tensor encoding (b = 2.8 ms/μm2) of a single slice from one representative animal in the retinal ischemia group. Linear, planar and spherical tensor encodings (LTE, PTE, STE) and a non-diffusion-weighted image (b = 0 ms/μm2) are shown. The yellow rectangle indicates the optic nerves. (TIF) [file pone.0282549.s002.tif]

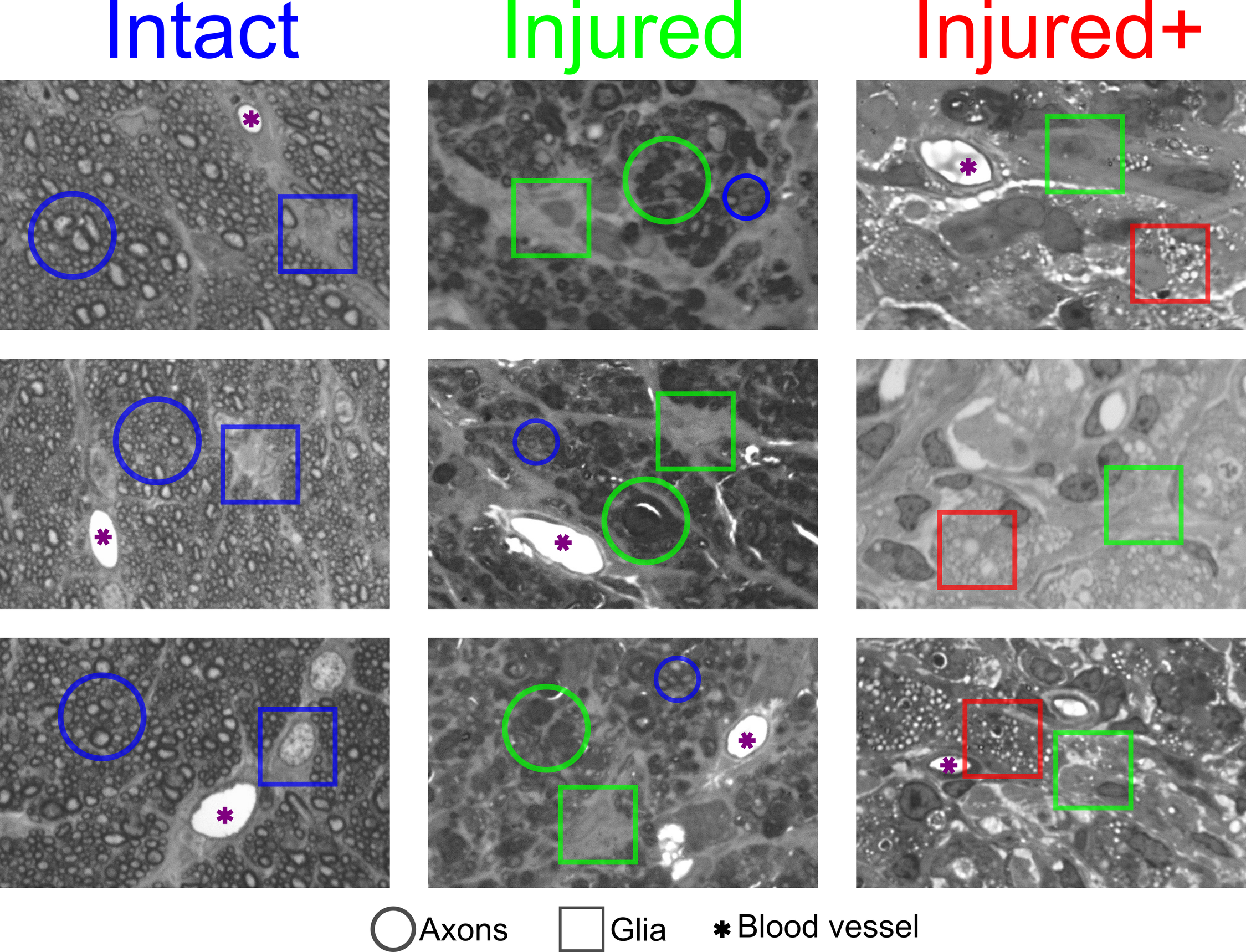

Supplement: S3 Fig — Photomicrograph samples of nine different specimens in the study. Columns share unique histological characteristics that represent each class. Intact: Densely-packed myelinated axons (blue circles), and normally appearing sharp, elongated glial processes (blue squares). Injured: Multiple collapsed axons (green circles) interspersed with small viable axons (small blue circles), surrounded by enlarged ameboid glial processes (green squares). Injured+: There is no axonal population left, with abundant large ameboid glial processes (green squares), many of which have foamy interiors (red squares). Purple asterisks correspond to blood vessels. (TIF) [file pone.0282549.s003.tif]

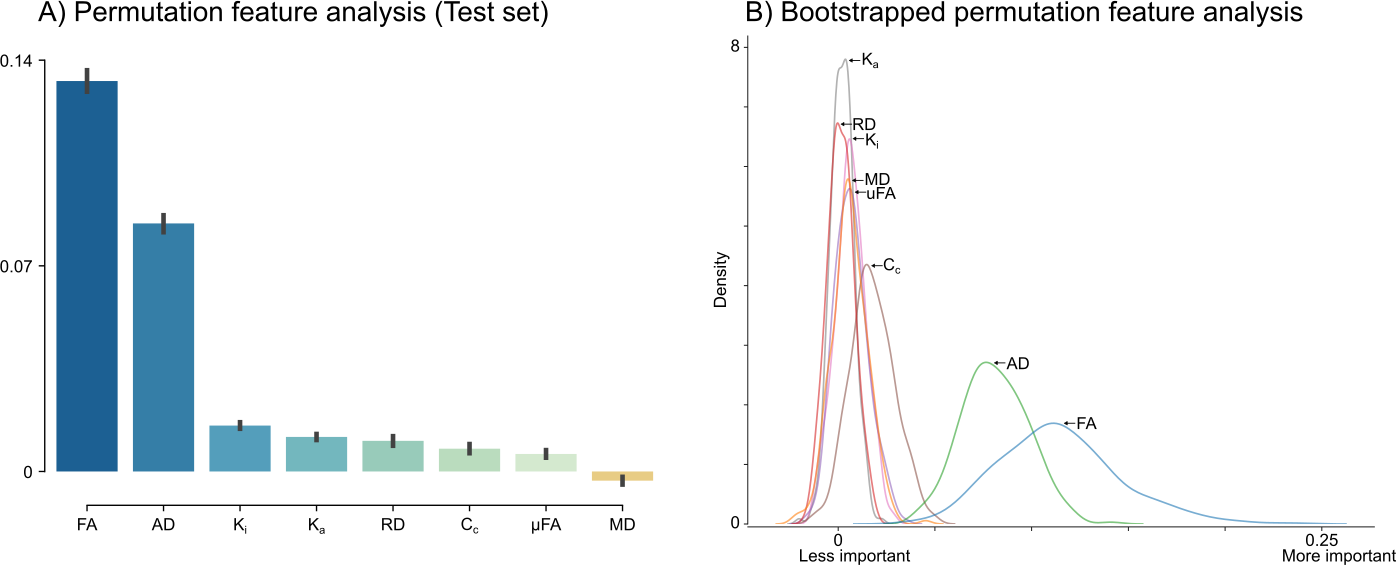

Supplement: S4 Fig — A) Permutation feature relevance analysis in the test set. B) Bootstrapped permutation feature analysis. FA and AD are the most important features. Gini importance (B) showed Cc as the third-ranking relevant feature. (TIF) [file pone.0282549.s004.tif]

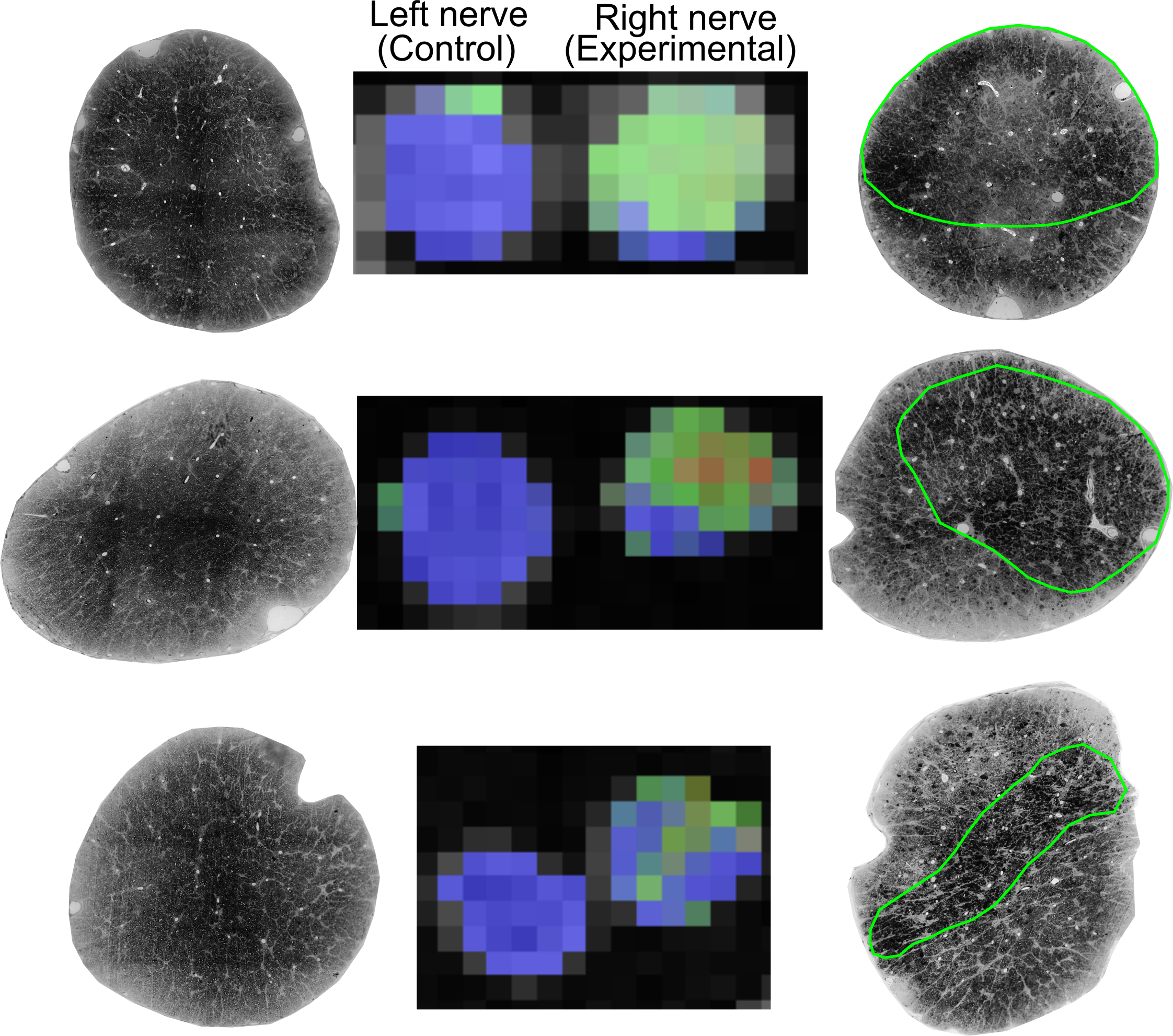

Supplement: S5 Fig — (TIF) [file pone.0282549.s005.tif]
